# The CCL2 synthesis inhibitor *bindarit* targets cells of the neurovascular unit, and suppresses experimental autoimmune encephalomyelitis

**DOI:** 10.1186/1742-2094-9-171

**Published:** 2012-07-12

**Authors:** Shujun Ge, Bandana Shrestha, Debayon Paul, Carolyn Keating, Robert Cone, Angelo Guglielmotti, Joel S Pachter

**Affiliations:** 1 Department of Cell Biology, Blood–brain Barrier Laboratory, 263 Farmington Ave., Farmington, CT, 06030, USA; 2 Department of Immunology, University of Connecticut Health Center, 263 Farmington Ave., Farmington, CT, 06030, USA; 3 Angelini R&D, Angelini Research Center, S. Palomba-Pomezia, Rome, 00040, Italy

**Keywords:** CCL2, Neuroinflammation, Blood–brain barrier, Neurovascular unit, Brain microvascular endothelial cells, Astrocytes, Microglia

## Abstract

**Background:**

Production of the chemokine CCL2 by cells of the neurovascular unit (NVU) drives critical aspects of neuroinflammation. Suppression of CCL2 therefore holds promise in treating neuroinflammatory disease. Accordingly, we sought to determine if the compound bindarit, which inhibits CCL2 synthesis, could repress the three NVU sources of CCL2 most commonly reported in neuroinflammation – astrocytes, microglia and brain microvascular endothelial cells (BMEC) – as well as modify the clinical course of neuroinflammatory disease.

**Methods:**

The effect of bindarit on CCL2 expression by cultured murine astrocytes, microglia and BMEC was examined by quantitative reverse transcription polymerase chain reaction (qRT-PCR). Bindarit action on mouse brain and spinal cord *in vivo* was similarly investigated by qRT-PCR following LPS injection in mice. And to further gauge the potential remedial effects of bindarit on neuroinflammatory disease, its impact on the clinical course of experimental autoimmune encephalomyelitis (EAE) in mice was also explored.

**Results:**

Bindarit repressed CCL2 expression by all three cultured cells, and antagonized upregulated expression of CCL2 in both brain and spinal cord *in vivo* following LPS administration. Bindarit also significantly modified the course and severity of clinical EAE, diminished the incidence and onset of disease, and evidenced signs of disease reversal.

**Conclusion:**

Bindarit was effective in suppressing CCL2 expression by cultured NVU cells as well as brain and spinal cord tissue *in vivo*. It further modulated the course of clinical EAE in both preventative and therapeutic ways. Collectively, these results suggest that bindarit might prove an effective treatment for neuroinflammatory disease.

## Background

The chemokine CCL2 (formerly called MCP-1) is a critical mediator of neuroinflammation in a myriad of diseases states, including multiple sclerosis (MS) and its animal model experimental autoimmune encephalomyelitis (EAE) [1], HIV-1 encephalitis [2], Guillain-Barré Syndrome [3], Alzheimer’s disease [4], ischemia [5], neurotrauma [6], epilepsy [7], neurogenic hypertension [8] and alcoholism [9]. While its precise mechanisms of action remain to be elaborated, among CCL2’s widely recognized effects are disruption of the blood–brain barrier (BBB) [10-12] and stimulated migration of mononuclear leukocytes into the central nervous system (CNS) [13-17].

These actions and pathogenic role, along with the fact that constitutive expression of CCL2 in the healthy central nervous system is severely limited [18], render CCL2 an ideal target for therapeutic intervention in neuroinflammatory disease [17,19,20]. Indeed, there is already strong suggestion that pharmacological suppression of CCL2 expression [21,22], oligomerization [23,24] or binding to its cognate receptor, CCR2 [25,26], can mitigate aspects of EAE. Pharmacologic blockade of CCL2 binding to glycosaminoglycans (GAGs) has also been reported to antagonize an autoimmune inflammatory condition of the neural retina, experimental autoimmune uveitis [27].

While highly effective in moderating neuroinflammation experimentally, many pharmacological agents that abrogate CCL2 expression and/or activity have nevertheless failed clinically. This disappointing performance in clinical trials might stem, in part, from overly broad suppression of microglia and astrocytes, a potential caveat that could curtail beneficial action of these cells in resolving neuroinflammation [28,29], as well as redundancy of chemokine binding sites and targets [30,31]. An alternative approach that more selectively targets CCL2 synthesis might therefore hold therapeutic promise in the treatment of human neuroinflammatory disease.

An attractive candidate in this regard is the well-characterized compound 2-((1-benzyl-indazol-3-yl) methoxy)-2-methyl propionic acid (bindarit) [32]. A small, synthetic indazolic derivative that preferentially inhibits transcription of the monocyte chemoattractant subfamily of CC chemokines (MCP-1/CCL2, MCP-2/CCL8 and MCP-3/CCL7) [33], bindarit has shown clinical efficacy in a broad array of experimental inflammatory, autoimmune and vascular disorders involving peripheral organ beds [34-38], as well as success in recent clinical trials for diabetic nephropathy [39] and lupus nephritis [40]. Such efficacy has been associated with bindarit’s ability to interfere with monocyte recruitment, which is also a critical feature in neuroinflammatory disease [13-17].

Given this clinical history of bindarit suppressing various examples of peripheral inflammation, we investigated its effect on expression of CCL2 in culture by the three cell types that represent the most frequently reported CNS sources of this chemokine during neuroinflammation: astrocytes, microglia and brain microvascular endothelial cells (BMEC). These cells serve as integral components of the neurovascular unit (NVU) [41] and, via their expression of CCL2, can impact the BBB and course of neuroinflammatory disease [42,43]. As a complement to these culture studies, the ability of bindarit to suppress LPS induction of CNS CCL2 expression *in vivo* was well determined. And to gauge bindarit’s potential clinical efficacy, its effect on EAE, a prototypical neuroinflammatory disease [44,45], was also examined. Results indicate bindarit significantly suppressed CCL2 gene expression in culture, as well as blunted lipopolysaccharide (LPS)-induced expression of CCL2 in the CNS. It also inhibited various facets of clinical EAE, and showed signs of promoting disease recovery. Collectively, these data suggest that bindarit might offer promise, either alone or in conjunction with other therapies, in the treatment of human neuroinflammatory disease.

## Methods

### Reagents

All reagents and antibodies were purchased from Sigma-Aldrich (St. Louis, MO, USA), unless specified otherwise. Bindarit was synthesized by and obtained from Angelini (Angelini Research Center-ACRAF, Italy). MOG peptide_35-55_ was synthesized by the WM Keck Biotechnology Resource Center at Yale University, New Haven, CT, USA.

### Preparation of bindarit

For experiments with cultured cells, a stock solution of 100 mM bindarit was prepared in dimethyl sulfoxide (DMSO), and dilutions (50, 100, 300 and 500 μM) of the DMSO stock were made in culture medium. For *in vivo* experiments, bindarit was prepared as a suspension in 0.5% methylcellulose (MTC) at a concentration of 20 mg/ml as previously described [37].

### Mice

C57BL/6 mice were obtained from the Charles River Laboratories, Inc. (Wilmington, MA, USA). All animal studies were performed, and CO_2_-mediated euthanasia carried-out, according to the Animal Care and Guidelines of the University of Connecticut Health Center (Animal Welfare Assurance #A3471-01).

### Isolation and culture of mouse astrocytes and microglia

Brain tissue obtained from mice at postnatal days 2 to 3 was used as the source of astrocytes and microglia. After decapitation, brains were removed immediately and separate astrocyte and microglial cultures prepared following a modified version of the protocol described by Ge and Pachter [46]. Cerebral cortices were first cut into small pieces (approximately 1 mm), and the minced tissue incubated in dissecting medium (Hank’s Balanced Salt Solution, from Gibco/BRL, Rockville, MD, USA), containing 0.5% glucose, 0.7% sucrose, 20 mM: hydroxyethyl piperazineethanesulfonic acid (Hepes) (pH 7.4) with 0.03% trypsin at 37 °C for 20 to 30 minutes. The tissue extract was then centrifuged at 1000 × g for 5 minutes and the resulting pellet washed and resuspended in astrocyte culture medium (Earl’s Modified Eagle Medium, from Gibco/BRL) containing 10% fetal bovine serum, 10% horse serum, 2 mM glutamine, 20 mM d-glucose, 4 mM sodium bicarbonate, 100 μg/ml penicillin and 100 μg/ml streptomycin. The tissue was mildly triturated to produce a single cell suspension, and the dissociated cells plated onto tissue culture flasks (T-75 cm^2^) coated with poly-lysine (BD Biosciences, Bedford, MA, USA). Cultures were maintained up to 1 week in plating medium in a humidified atmosphere (5% CO_2_) at 37 °C. After this time, cultures were shaken at 200 rpm for 2 hr at 4 °C, and supernatants containing dislodged microglia collected. Supernatant material was then centrifuged at 1000 × g for 5 minutes to pellet microglia. Microglia were then resuspended in microglia culture medium (Dulbecco’s modified Eagle Medium, from Gibco-BRL) supplemented with 10% heat-inactivated fetal calf serum, 100 μg/ml penicillin and 100 μg/ml streptomycin) and cultured in a 24-well plate. Following removal of microglia from the initial mixed glial cultures, the latter were shaken for an additional 18 hr at 37 °C to remove residual neurons. The enriched astrocyte population that remained was further depopulated of remaining microglia by treatment with L-leucine methyl ester (LME) [47]. LME was dissolved in astrocyte culture medium, and the solution adjusted to pH 7.4 and filtered prior to adding to cultures to achieve a final concentration of 50 mM. After 90 minutes of LME treatment, astrocyte-enriched cultures were washed thoroughly and re-incubated with fresh astrocyte culture medium. Cell purity was determined by immunocytochemistry using a monoclonal anti-human glial fibrillary acid protein (GFAP) antibody, and cultures assessed to be ≥ 98% astrocytes (GFAP+).

### Isolation and culture of mouse brain microvascular endothelial cells

BMEC were isolated as previously detailed by this laboratory [10,48]. Primary cultures were typically grown for approximately five days prior to sub-culturing for experiments. At that time, purity was gauged to be ≥ 98% BMEC, according to diI-acetylated LDL uptake [48]. BMEC also exhibited common endothelial characteristics, e.g. CD31 and vWF immunostaining, plus displayed expression of the tight junction-associated proteins ZO-1 and occluding, found enriched at the BBB.

### Treatment of cultured cells

To examine effects of bindarit on basal CCL2 expression, cultured microglia and BMEC were incubated with different concentrations of bindarit for 4 hr or exposed to 300 μM bindarit for different time. To gauge effects of bindarit on LPS-stimulated CCL2 expression, astrocytes and microglia were pretreated with 300 μM bindarit for 1 hr; then cells were incubated with ± 100 ng/ml LPS (*Escherichia coli* Serotype 026:B6) for 4 or 20 hr in the continued presence of bindarit. After treatments, cells were extracted for RNA purification.

### Separation of brain microvessels and parenchyma

Distinct brain microvessel and parenchymal fractions were obtained using a modification of the method to prepare BMEC [10,48]. In brief, after removal of the brain from the cranium, the meninges and big vessels were discarded, and the whole brain diced into approximately 1 mm pieces. Brain tissue was then homogenized with a 7 mL Dounce tissue grinder (Kimble/Kontes, Vineland, NJ, USA) in PBS. The homogenate was then transferred to a 15 ml conical tube and centrifuged at 400 × g for 15 minutes in an Eppendorf Model 5804R centrifuge/A-4-44 rotor. The resulting pellet was resuspended in 18% dextran (v/v, molecular weight 60 000 to 90 000; USB Corporation, Cleveland, OH, USA) and centrifuged at 4,500 × g for 15 minutes to pellet the crude microvessel fraction. The dextran supernatant and floating layer of myelinated axons were separated from the crude microvessel pellet, then diluted 1:2 with PBS and centrifuged at 720 × g for 10 minutes to sediment the parenchymal fraction. Both microvessel and parenchymal fractions were washed twice with PBS to eliminate traces of dextran. Microvessels were further washed free of blood cells by filtering through a 40 μm cell strainer (Becton Dickinson Labware, Franklin Lakes, IN, USA) and eluting with PBS. Eluted microvessels were pelleted by centrifugation at 16,000 × g in a table-top microcentrifuge.

### Treatment of animals

To determine effects of bindarit on LPS-stimulated CCL2 expression in brain and spinal cord, C57BL/6 female mice were given intraperitoneal (i.p.) injection of bindarit (200 mg/kg) or methylcellulose vehicle, once a day, for 4 consecutive days. At 30 minutes following the last bindarit injection, mice were given i.p. injection of LPS (5 mg/kg; *Escherichia coli* Serotype 026:B6). Then, 4 hr after LPS injection, mice were euthanized and brain and spinal cord dissected for CCL2 mRNA and protein analysis.

For active induction of EAE, C57BL/6 female mice were immunized with MOG_35-55_ peptide (MEVGWYRSPFSRVVHLYRNGK) of rat origin, by a modification of the method previously described [49]. Briefly, on day 0 female mice 7 to 9 weeks of age were injected subcutaneously with 150 μg of MOG peptide and 300 μg of *Mycobacterium tuberculosis* (DIFCO, Detroit, MI, USA) in complete Freund’s adjuvant (CFA) (DIFCO) into the right and left flank, 100 μl per site. Mice were also injected i.p. with 200 ng pertussis toxin (List Laboratories, Campbell CA, USA) in PBS on days 0 and 2 following the first immunization. Animals were monitored and scored daily for clinical disease severity according to the following scale: 0 = normal; 1 = tail limpness; 2 = limp tail and weakness of hind legs; 3 = limp tail and complete paralysis of hind legs; 4 = limp tail, complete hind leg and partial front leg paralysis; and 5 = death. Several disease parameters were calculated as described [49]. The mean day of onset was calculated by averaging the time when clinical symptoms, that is, a clinical score ≥ 1, were first observed for two consecutive days in each mouse. The mean maximum clinical score was calculated by averaging the highest score for each mouse. The disease index was calculated by adding the daily average clinical scores in each group, dividing by the mean day of onset, and multiplying by 100. In the case that an animal showed no disease, the day of onset was arbitrarily counted as one day after the last day of the experiment (for example, day 22). And the disease incidence was the fraction of mice experiencing EAE.

To investigate the effects of bindarit on both the clinical course of EAE and CCL2 level during disease, mice were given daily i.p. injection of bindarit (or vehicle MTC) at 200 mg/kg for three consecutive days, beginning the day before MOG immunization (day −1), then injections every other day till day 20. This schedule was designed to mitigate, as much as possible, trauma associated with daily injections at times of peak neurologic disease and physical compromise.

### RNA purification from cell cultures

Total RNA was extracted from cell cultures using the RNeasy kit according to the manufacturer’s instructions. RNA was treated with Turbo DNAse (Ambion, Austin, TX, USA) according to the protocol provided by the manufacturer. RNA yield and purity were determined by spectrophotometry absorption at 260 and 280 nm.

### RNA purification from CNS tissue

RNA and protein were differentially extracted from the same mouse brain and spinal cord samples using the AllPrep RNA/Protein kit (QIAGEN, Valencia, CA) following the manufacturer’s instructions. RNA was treated with Turbo DNAse (Ambion, Austin, TX, USA) according to the protocol provided by the manufacturer. RNA yield and purity were determined by spectrophotometry absorption at 260 and 280 nm. Protein level was determined using the Micro BCA protein assay kit (Pierce, Rockford, IL, USA), using bovine serum albumin as a standard.

### Reverse transcription

cDNA was synthesized from the total RNA using a SuperScript III (Invitrogen, Carlsbad, CA, USA) First-strand synthesis system for RT-PCR with a standard protocol. The resulting cDNA was stored at −80 °C until used for further analysis.

### CCL2 RNA determination by quantitative RT-PCR

Measurements of cDNA levels were performed by quantitative (q) RT-PCR using an ABI PRISM 7500 Sequence Detection System Version 1.3, and SYBR green (AB Applied Biosystems, Foster sity, CA, USA) fluorescence was used to quantify relative amplicon amount. Separate controls included a no template-control and no reverse transcriptase-control, and standard curves were constructed for all primers used. Cycle time (Ct) values for all samples were normalized to RPL-19, the housekeeping gene encoding the 60 S ribosmal protein L19. Specifically, relative amplicon quantification was performed using the formula: $\left(1+\text{Eref}\right)\text{Ct}\left(\text{ref}\right)/\left(1+\text{Etarget}\right)\text{Ct}\left(\text{target}\right)\times \;100\%$, with ref: RPL19; target: CCL2; E: primer pair efficiency; and Ct: threshold cycle.

For all cell culture studies and *in vivo* LPS studies, relative CCL2 gene expression values (after normalization to RPL19) were expressed as percentage of control. For EAE studies, relative CCL2 gene expression values were designated as percentage of RPL-19 expression, as control CCL2 level (time-point 0) was undetectable. The primer sequences used in this study were as following: for mouse CCL2, 5′- GGC TCA GCC AGA TGC AGT TAA-3′ (forward) and 5′- CCA GCC TAC TCA TTG GGA TCA −3′ (reverse); for RPL-19, 5′- CGC TGC GGG AAA AAG AAG-3′ (forward) and 5′- CTG ATC TGC TGA CGG GAG TTG −3′ (reverse).

### CCL2 protein determination

The level of CCL2 was measured with mouse JE/CCL2 commercial enzyme-linked immunoassay kit (BioSource International Inc., Camarillo, CA) according to the manufacturer’s instructions.

### Statistical analysis

Statistical significance of differences between mean values of bindarit-treated cultures and control cultures was analyzed using a paired two-tailed *t*-test, while comparisons of bindarit treatment on LPS-treated mice were performed using a two-tailed *t*-test for independent samples. For analysis of bindarit effects on clinical EAE, a *chi*-squared (*χ*^2^) test was used for comparisons of disease incidence; a Mann–Whitney *U*-test was used for comparisons of disease index; and a two-tailed *t*-test for independent samples was used for comparison of disease onset. A *P*-value < 0.05 was considered significant in all cases.

## Results

### Bindarit differentially suppresses CCL2 expression by cultured CNS cells

**Figure 1:**
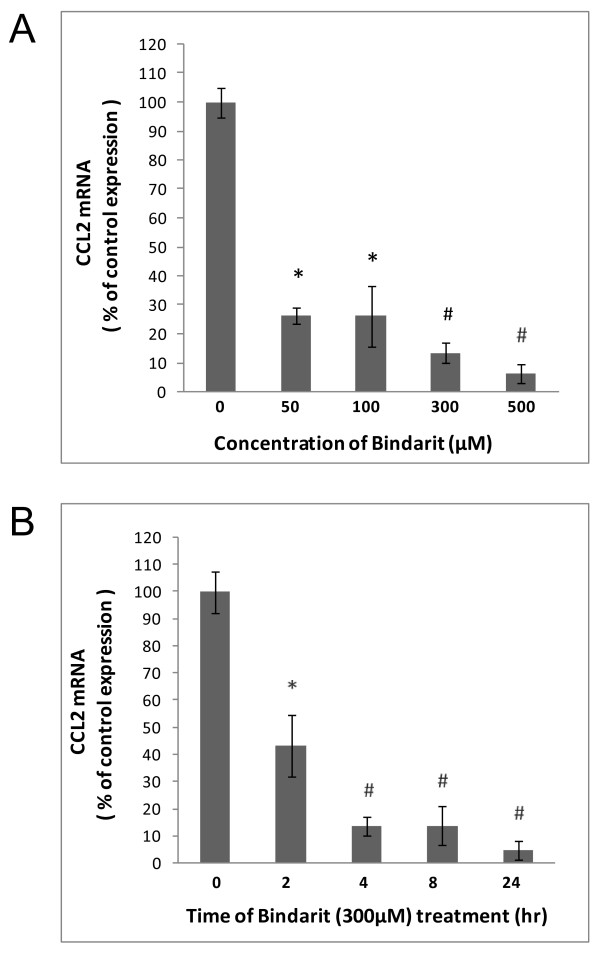
**Bindarit effects on CCL2 mRNA in cultured microglia.** Microglia, prepared from C57BL/6 mouse brain, were incubated with bindarit at the indicated concentrations for 4 hr **(A)**, or exposed to 300 μM bindarit for the indicated times **(B)**. Control cultures received only vehicle. Relative CCL2 RNA levels were determined by qRT-PCR, and effects of bindarit treatment reported as % change compared to control cultures. *P < 0.05; ^#^*P* < 0.01 (compared to control at 0 concentration or 0 time of bindarit).

**Figure 2:**
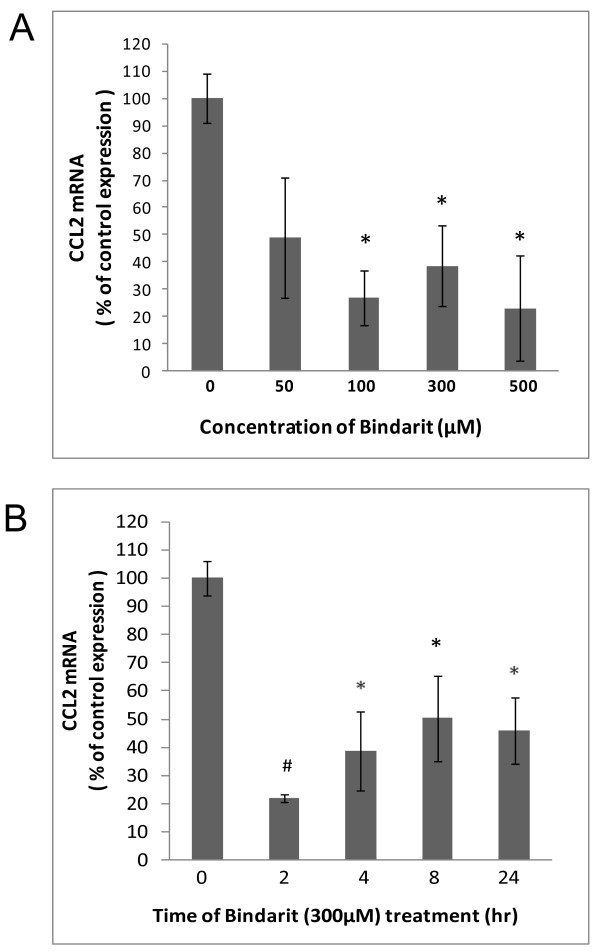
**Bindarit effects on CCL2 mRNA in cultured BMEC.** Brain microvascular endothelial cells (BMEC), prepared from C57BL/6 mouse brain, were incubated with bindarit at the indicated concentrations for 4 hr **(A)**, or exposed to 300 μM bindarit for the indicated times **(B)**. Control cultures received only vehicle. Relative CCL2 RNA levels were determined by qRT-PCR, and effects of bindarit treatment reported as % change compared to control cultures. *P < 0.05; ^#^*P* < 0.01 (compared to control at 0 concentration or 0 time of bindarit).

**Figure 3:**
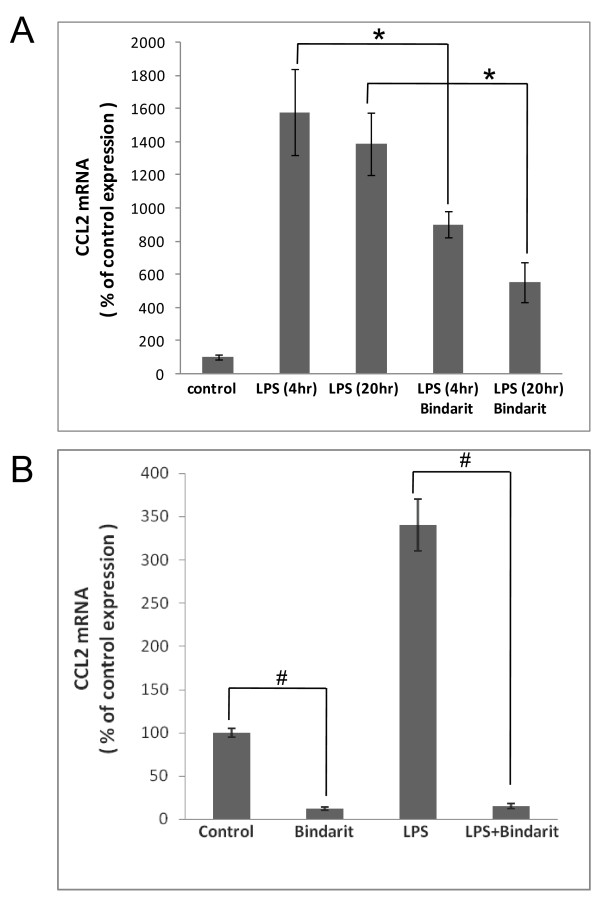
**Bindarit effects on LPS-stimulated CCL2 mRNA in cultured astrocytes and microglia.** Separate cultures of astrocytes **(A)** and microglia **(B)**, prepared from C57BL/6 mouse brain, were pre-treated with 300 μM bindarit for 1 hr, then incubated with ± 100 ng/ml lipopolysaccharide (LPS) in the continued presence of bindarit. Astrocytes cultures were exposed to LPS for the indicated times to stimulate CCL2 expression. Microglial cultures were only exposed to LPS for 4 hr, as these highly express CCL2 even under basal conditions. Control cultures received only vehicle. Relative CCL2 RNA levels were determined by qRT-PCR, and effects of bindarit treatment reported as % change compared to control cultures. Brackets designate comparison between the two respective groups. *P < 0.05; ^#^*P* < 0.01.

The effects of bindarit on cultured glial and BMEC were investigated first (Figures 1, 2, 3). Figure 1 shows that cultured microglia demonstrated both a dose and time dependency of bindarit effect on CCL2 mRNA level. Suppression of basal CCL2 mRNA was seen beginning with the lowest dose of 50 μM for 4 hr, amounting to nearly 75% reduction. Increasing the dose to 300 and 500 μM resulted in still further diminution of CCL2 mRNA to approximately 10% and 5%, respectively, of control level. Treatment with bindarit at 300 μM for as little as 2 hr resulted in near 60% reduction in CCL2 mRNA level, and treatment for longer times at this concentration resulted in suppression of CCL2 mRNA to ≥ 90% of control level.

BMEC demonstrated a similar qualitative response in basal CCL2 mRNA to increasing bindarit concentration, but suppression was not as severe as seen with microglia (Figure 2). Significant reduction was not observed until 100 μM, and the maximal suppression achieved was about 20% that of control. The time course of bindarit action on BMEC also differed. Maximal suppression by 300 μM bindarit was achieved at the earliest time-point of 2 hr, reaching a level of approximately 20% of that of the control. Longer time-points, however, appeared to result in a lesser effect. It is important to reemphasize that, in the normal CNS, CCL2 expression is barely detectable. This would suggest that both cultured microglia and BMEC, possibly removed from a normally suppressive microenvironment, are in a somewhat activated state.

This situation appears different for astrocytes. In this case, bindarit’s effects on constitutive CCL2 gene expression could not be accurately assessed, as level of this chemokine’s mRNA in murine culture of these cells is very low. Astrocyte cultures were thus stimulated with LPS for different lengths of time to greatly induce CCL2 mRNA, and the effect of bindarit on this CCL2 induction was assayed (Figure 3A). Stimulation with 100 ng/ml LPS for both 4 hr and 20 hr produced similar elevations in CCL2 gene expression, and bindarit treatment at 300 μM similarly suppressed, by 40 to 60%, the induction of CCL2 mRNA at both time-points.

In light of bindarit’s success at mitigating induction of CCL2 in astrocytes, we next assayed whether it was similarly effective in preventing induction in LPS-stimulated microglia. Figure 3B shows that this was in fact the case, bindarit completely suppressing the induction and reducing CCL2 mRNA level to 15% of the control (basal) value.

### Bindarit blocks LPS-induced CCL2 expression in brain and spinal cord

**Figure 4:**
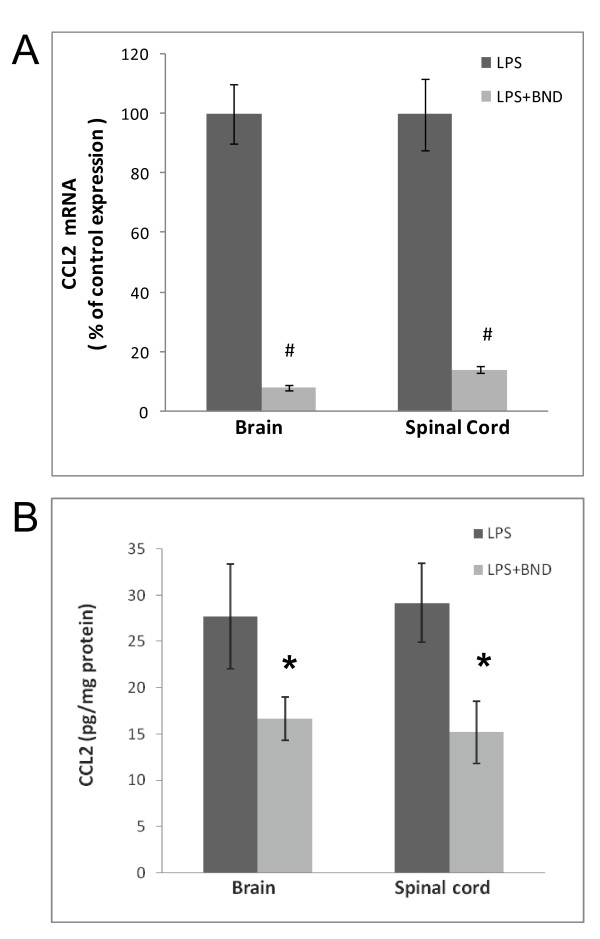
**Bindarit’s effects on CCL2 mRNA and protein in brain and spinal cord following LPS.** Mice received i.p. injection of 200 mg/kg bindarit (or vehicle) once a day for 4 days, followed by i.p. injection of 5 mg/kg lipopolysaccharide (LPS) at 30 minutes after the last bindarit treatment. At 4 hr after LPS injection, brain and spinal cord were prepared for CCL2 mRNA **(A)** and protein **(B)** determinations. LPS + bindarit-treated brain or spinal cord samples were compared to corresponding samples treated with LPS + vehicle (control). *P < 0.05; ^#^*P* < 0.01.

It was next investigated whether bindarit could suppress LPS-induced CCL2 expression in the CNS *in vivo* (Figure 4). In the normal, resting state, CNS CCL2 mRNA level is barely detectable in C57BL/6 mice [50], but is elevated 50- to 100-fold shortly after peripheral LPS injection [51]. Pretreatment with bindarit was nevertheless able to effectively block this induction both in the brain and spinal cord, by 92% and 86%, respectively. In addition to abrogating LPS-induction of CCL2 mRNA in the CNS, bindarit was also effective at reducing CCL2 protein level in both brain and spinal cord, though not to the same extent as mRNA.

### Bindarit therapeutically modifies clinical EAE

**Figure 5:**
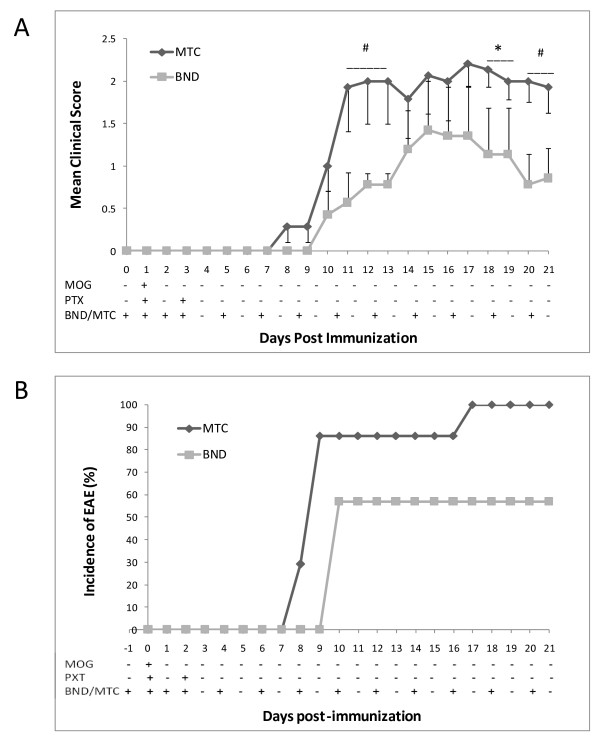
**Bindarit effects on clinical experimental autoimmune encephalomyelitis (EAE).** Mice were subject to EAE by active immunization with MOG peptide, beginning on day 0, as detailed in Methods. Bindarit (or vehicle) was injected i.p. at 200 mg/kg according to the schedule indicated, beginning at day −1 (one day before MOG immunization). Mean clinical score **(A)** and % incidence of EAE **(B)** were determined. EAE was diagnosed when animals demonstrated a clinical score ≥ 1 for two consecutive days.

The ability of bindarit to modify clinical EAE was examined next. Figure 5 shows that, with a bindarit schedule of daily injections for the first three days, and beginning at day −1, then injections every other day till day 20, bindarit yielded several therapeutic effects. By the criterion that disease is manifest when a clinical score of at least 1 is demonstrated for at least two consecutive days [49], bindarit delayed onset of EAE. Specifically, control mice developed acute disease beginning at day 8, while bindarit-treated mice did not show evidence of clinical disease until days 14 to 15. A second therapeutic effect observed was reduced disease progression and severity. Control mice showed rapid progression of EAE, proceeding towards a maximum mean clinical score of approximately 2.1 to 2.2 by day 9. Bindarit-treated mice evidenced slower progression, and only reached a maximum mean clinical score of 1.5. A third therapeutic effect was apparent reversal of disease course. After experiencing rapid onset, control mice showed a plateau in disease score typical of this monophasic MOG-induced EAE [52,53]. However, in marked distinction, bindarit-treated mice demonstrated a consistent downward trend in disease score following their delayed and attenuated peak in clinical presentation. A summary of the effects of bindarit treatment on clinical EAE is presented in Table 1.

**Table 1:** Summary of bindarit effects on clinical experimental autoimmune encephalomyelitis (EAE) parameters

**Group**	**Sick/total, number**	**Mean day of onset**^a^	**Mean max clinical score**	**Mean disease index**^b^
MTC	14/14	11.42 ± 1.76	2.43 ±0.47	163.7 ± 20.53
BND	8/14	16.85 ± 2.17	1.52 ± 0.88	99.3 ± 10.21
*P*-value	-	<0.005	<0.001	<0.01

Mean presented ± SD.

^**a**^Day of onset established when clinical score ≥ for two consecutive days.

^b^Disease index calculated at day 21.

**Figure 6:**
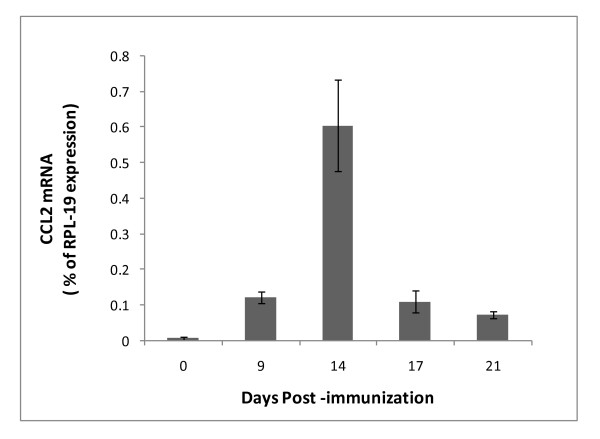
**CCL2 expression profile during experimental autoimmune encephalomyelitis (EAE).** Mice were immunized with MOG peptide to induce EAE. At the indicated days post-immunization, mice were sacrificed and CCL2 mRNA levels determined in the whole brain. CCL2 expression is seen to rapidly rise and fall between days 9 to 21, showing the highest level at day 14.

**Figure 7:**
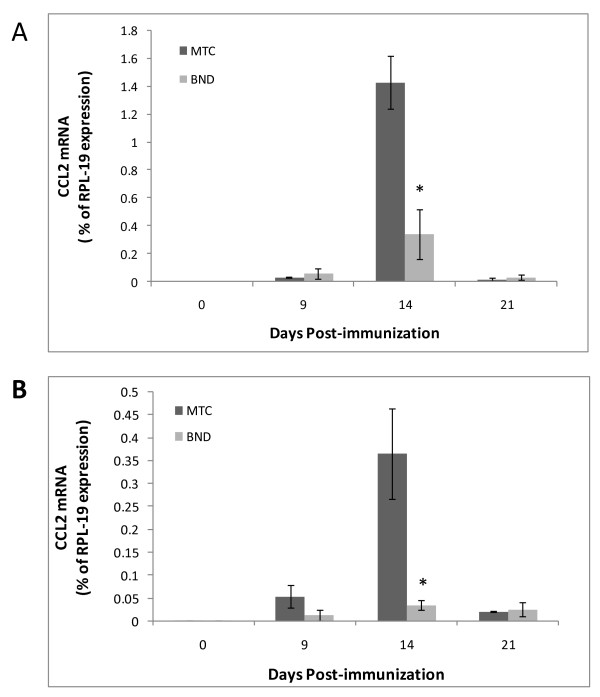
**Effects of bindarit on CCL2 expression in central nervous system (CNS) fractions during experimental autoimmune encephalomyelitis (EAE).** Mice were immunized with MOG peptide to induce EAE, and were injected with bindarit (or MTC vehicle) as in Figure 5. At the indicated days post-immunization, mice were sacrificed and CCL2 mRNA levels determined in microvascular **(A)** and parenchymal **(B)** fractions. Bindarit treated groups were compared to vehicle treated groups. *P < 0.01.

We next sought to examine how bindarit modifies CCL2 expression in the brain during EAE. First, the temporal expression of CCL2 was determined only in MOG-immunized mice not receiving any bindarit, to gauge the window of opportunity during which bindarit might act. As seen in Figure 6, CCL2 RNA is barely detectable at the time of immunization. Its expression then accelerates beginning around day 9, is significantly elevated by day 14, rapidly declines at day 17, and reaches near basal level by day 21. Because bindarit has been shown to most effectively suppress stimulated, rather than basal, CCL2 expression [33,54], bindarit effects on CCL2 were analyzed selectively during this peak interval. Additionally, brain tissue was resolved into microvascular and parenchymal fractions to further identify targeted cell types. This resolution was deemed important, as both microvessels (BMEC) and parenchymal neural cells (astrocytes and microglia) have been reported as sources of CCL2 during EAE [55-58], though microvessels only contribute < 1% to brain volume [59]. It was thus reasoned that parenchymal effects could overshadow possible bindarit-induced changes in microvascular CCL2 expression if only whole-brain levels were evaluated. Figure 7 reveals that bindarit significantly reduced peak CCL2 expression during EAE in both microvascular and parenchymal fractions, in agreement with what was found in our culture studies. Also, bindarit did not affect CCL2 expression outside the peak window, reinforcing that its action appears restricted to activated cells within and outside the CNS [33,54].

## Discussion

Given the success of the CCL2 synthesis inhibitor bindarit in ameliorating several animal disease models and human clinical conditions associated with peripheral inflammatory disease, initial studies were conducted to examine its effects on elements critical to neuroinflammatory disease. Focusing on the three main cell types responsible for CCL2 expression during neuroinflammation, experiments revealed bindarit significantly suppressed CCL2 in cultured BMEC, microglia and astrocytes. Bindarit was further shown to be effective *in vivo* in two neuroinflammatory paradigms: 1) it blocked LPS induction of CCL2 in both brain and spinal cord; and 2) it therapeutically modified the course of EAE while suppressing CCL2 expression in both brain microvascular and parenchymal compartments.

As to the effects on the seminal sources of CCL2, it was critical to determine whether each was susceptible to bindarit, as the specific cellular pool(s) responsible for CCL2’s pathogenic actions during neuroninflammatory disease remain unclear [51]. While all three cell types responded with significant reduction in CCL2 mRNA, microglia were the most sensitive - experiencing > 90% diminution in this chemokine’s expression. This high sensitivity to bindarit holds particular significance, as microglia are widely considered the primary immune effector cells in the CNS [60-63], and their expression of CCL2 has been linked to monocyte recruitment into the CNS [64,65]. As CCL2 can also direct recruitment and proliferation of microglia [66-68], as well as activation of these cells [68], microglial expression of CCL2 can potentially support a self-sustaining cycle of neuroinflammation. Bindarit action, however, might effectively abrogate such a scenario.

That bindarit also suppressed CCL2 mRNA in BMEC is noteworthy. As these cells form the first line of defense in the BBB [69], their expression of CCL2 might strongly influence incipient steps of neuroinflammation [70]. Indeed, elevated CCL2 expression by BMEC has been reported in MS [71] and EAE [55,56], as well as in autoimmune inflammation of the peripheral nervous system [3]. Furthermore, intravenously administered anti-CCL2 antibody blocked heightened leukocyte adhesion to pial venular endothelium *in vivo* in mice suffering acute EAE [72], as well as prevented recurring clinical episodes in a chronic relapsing EAE model [73], possibly by antagonizing CCL2 at the luminal endothelial surface. Supporting this possibility, CCL2 harbors in its C-terminal α-helix a binding site for GAGs typically found on the luminal endothelial surface [74], and has been shown to bind to the luminal surface of cultured endothelial cells and then trigger firm adhesion followed by transmigration of mononuclear leukocytes [75,76]. Binding of CCL2 released from BMEC in culture has most recently been shown to switch from the luminal to the abluminal surface following cytokine-induced activation [77], possibly reflecting the changing roles of this chemokine pool from first promoting leukocyte adhesion to later directing extravasation into the parenchyma. Thus, by targeting the BMEC reservoir of CCL2 during disease, bindarit might be able to blunt neuroinflammation at different stages.

Bindarit action on CCL2 expression by cultured astrocytes had to be studied in the context of LPS stimulation, as these cells exhibit barely detectable CCL2 mRNA in culture or *in situ* in the naïve state [50,51]. Yet despite significant induction, astrocyte CCL2 mRNA was reduced by half or more following bindarit exposure. As astrocytes constitute the most abundant glial cell population in the CNS [78], suppression of their CCL2 production by bindarit *in vivo* might well exert profound influence on pathologic events.

That bindarit could indeed act *in vivo* to effectively suppress neuroinflammation was evident in both the LPS and EAE paradigms. Injection of bindarit dramatically reduced LPS-stimulated expression of CCL2 in both brain and spinal cord, dropping mRNA levels to near 10% of vehicle-injected control values, while cutting protein levels approximately by half. In this case, the efficacy of bindarit in suppressing brain CCL2 may have been aided by the fact that LPS can severely disrupt the BBB [79,80], and thereby possibly facilitate bindarit entry into the CNS parenchyma. As CCL2 can also disrupt tight junctions leading to elevated BBB permeability [10-12], CCL2 generated early after LPS injection may have contributed to its subsequent suppression by further enabling bindarit CNS access.

The effects of bindarit on clinical EAE suggest that bindarit exerted both preventative and therapeutic actions. Preventative action is indicated by the considerable delay in disease onset in the bindarit-treated group, as well as the reduced incidence and severity of disease displayed by these mice. Possible therapeutic action is conveyed by the steady decline in disease severity following diminished peak clinical score. Such decline was in marked contrast to the typical plateau in clinical score exhibited by EAE mice given vehicle. These results are qualitatively similar to those recently reported by Laborde *et al.*[81] who, employing a regimen of twice daily oral dosage of a novel heteroaroylphenylurea antagonist of CCL2 function, also described delayed disease onset and resolution of EAE symptoms. In the case of bindarit, however, clinical symptoms seemed to steadily remit following attenuated peak disease, and a reduced incidence was also noted. Both studies nevertheless highlight the prospect that selective targeting of CCL2 activity might prevent EAE, as well as reverse its course.

The effect of bindarit on clinical EAE was accompanied by significant reduction of CCL2 mRNA in both brain microvessel and parenchymal fractions, consistent with bindarit’s mechanism of action being inhibition of CCL2 transcription [33]. Reinforcing this point, global knockout of the CCL2 gene has been shown to similarly delay EAE onset, and reduce both disease incidence and severity, effects that have been attributed to absence of CCL2 expression within the CNS compartment [81,82]. This, along with demonstration that CCL2-deficient mice also exhibit reduced neuroinflammatory responses to peripheral LPS injection [83,84], underscores CCL2’s non-redundant role in neuroinflammatory disease and accentuates its value as a therapeutic target. Results with bindarit and EAE may further suggest that both microvascular and parenchymal sources of CCL2 contribute to pathogenesis. If this is so, it could further imply bindarit would not have to penetrate the BBB in order to reach at least one of its targets, BMEC. In contrast to the acute situation with LPS, which acts directly on the endothelium, it is reasoned that the BBB was more intact in EAE mice receiving bindarit, as mean disease score only reached approximately 1.5. Thus, a likely scenario is that bindarit also sufficiently crossed the BBB to suppress the astrocyte and/or microglial response as well. This lends promise that bindarit can access the CNS parenchyma during the early stages of neuroinflammatory disease, when BBB breakdown is not yet manifested.

## Conclusions

In summary, the CCL2 synthesis inhibitor bindarit, previously shown to be highly effective in myriad experimental disease models as well as human conditions having inflammatory involvement [34-40], was observed to significantly reduce steady state and LPS-induced CCL2 expression in cultured microglia, BMEC and astrocytes, as well as LPS-stimulated CCL2 mRNA and protein levels in CNS tissue *in situ*. Bindarit was further effective in delaying, preventing and attenuating clinical EAE, and evidenced signs of possibly reversing disease course while also suppressing elevation of CCL2 in brain microvascular and parenchymal compartments. Collectively, these data are consistent with the widely proposed critical role for CCL2 in neuroinflammation [18-20], and suggest bindarit, by targeting cells of the NVU [41], might have therapeutic success in the treatment of MS and/or other human neuroinflammatory diseases.

## Abbreviations

BBB, Blood–brain barrier; BMEC, Brain microvascular endothelial cells; CFA, Complete Freund’s adjuvant; CNS, Central nervous system; Ct, Cycle time; DMSO, Dimethyl sulfoxide; EAE, Experimental autoimmune encephalomyelitis; GAG, Glycosaminoglycan; GFAP, Glial fibrillary acid protein; Hepes, Hydroxyethyl piperazineethanesulfonic acid; i.p., Intraperitoneal; LME, L-leucine methyl ester; LPS, Lipopolysaccharide; MTC, Methylcellulose; MS, Multiple sclerosis; NVU, Neurovascular unit; PBS, Phosphate buffered saline; PCR, Polymerase chain reaction; RT, Reverse transcription.

## Competing interests

The author(s) declare that they have no competing interests.

## Authors' contribution

SG, BS, DP and CK performed all the experiments. SG assisted with design of the experiments and data analysis, prepared the figures, and participated in drafting the manuscript. AG and RC assisted with the data analysis. JP designed the experiments and wrote the manuscript. All authors read and approved the final manuscript.
